# Differential association of the 5-factor modified frailty index with postoperative pulmonary complications: specific prediction of infection risk after pulmonary lobectomy

**DOI:** 10.3389/fmed.2026.1771113

**Published:** 2026-04-13

**Authors:** Qianying Zheng, Zhuangbo Wu, Lingbo Du, Aiyang Chen, Jingmei Zhou, Jingxin Shi, Yuqing Lai, Wenbo Chen, Weiqi Ke, Huali Xu

**Affiliations:** 1 Department of Anesthesiology, The First Affiliated Hospital of Shantou University Medical College, Shantou, Guangdong, China; 2 Department of Anesthesiology, Jieyang Ciyun Hospital, Jieyang, Guangdong, China; 3 Shantou University Medical College, Shantou, Guangdong, China; 4 Department of Anesthesiology, Jieyang Haoze Hospital, Jieyang, Guangdong, China; 5 Department of Anesthesiology, People’s Hospital of Shantou, Shantou, Guangdong, China; 6 Department of Anesthesiology, Nanfang Hospital, Southern Medical University, Guangzhou, Guangdong, China

**Keywords:** frailty, lobectomy, modified frailty index, pleural effusion, pneumothorax, postoperative infection

## Abstract

**Background:**

Frailty is recognized as an important predictor of postoperative outcomes, yet evidence on the differential association between the 5-factor modified frailty index (mFI-5) and specific postoperative pulmonary complications (PPCs) following pulmonary lobectomy remains limited. This study aims to investigate the association between mFI-5 and the risk of distinct PPCs, with comprehensive adjustment for potential confounders.

**Methods:**

In this retrospective study of 391 patients undergoing lung resection, participants were stratified into Robust, Pre-frail, and Frail groups by mFI-5 score. The primary outcomes were postoperative pulmonary infection, pneumothorax, and pleural effusion, defined according to EPCO criteria with thoracic surgery-specific refinements. Multivariable logistic regression was used to assess associations, adjusting for comprehensive confounders. Multicollinearity was assessed using variance inflation factor (VIF). Subgroup analyses and interaction tests were performed.

**Results:**

Among the cohort (mean age 62.17 ± 8.71 years, 61.13% male), frailty status was associated with older age and a higher comorbidity burden. In fully adjusted models, the mFI-5 was a strong, independent predictor of postoperative pulmonary infection. Compared to the Robust group, the Pre-frail and Frail groups had significantly increased odds, with adjusted odds ratios (ORs) of 3.5 (95% CI: 1.6–7.9; *P* = 0.002) and 7.3 (95% CI: 2.0–26.2; *P* = 0.002), respectively. In contrast, no significant association was found between frailty and postoperative pneumothorax or pleural effusion (all *P* > 0.05). Subgroup analyses suggested that the risk of infection might be more pronounced in males and smokers, and that smoking history and surgical site might modify the associations with pneumothorax and effusion, respectively.

**Conclusion:**

The 5-factor modified frailty index (mFI-5) was independently associated with an increased risk of postoperative pulmonary infection, but not with pneumothorax or pleural effusion, following lung surgery. These findings suggest that the predictive capacity of mFI-5 varies across complication types.

## Introduction

Lung cancer, a type of malignant tumor originating in the bronchi, is globally recognized for its high incidence and mortality rates. In China, lung cancer ranks as the leading cause of both new cases and deaths among all types of malignancies. In 2022, approximately 1.06 million new cases of lung cancer were diagnosed in China, accounting for 22.0% of all new cancer diagnoses that year (1). Surgical resection combined with adjuvant chemoradiotherapy remains the cornerstone of early-stage lung cancer management. In recent clinical practice, video-assisted thoracic surgery (VATS) has been increasingly adopted as a minimally invasive alternative to conventional thoracotomy, demonstrating superior outcomes due to advantages including minimized trauma, reduced postoperative pain, and accelerated recovery (2).

Despite technological advancements, VATS lobectomy continues to be associated with a range of postoperative complications (3). Among these, postoperative pulmonary complications (PPCs) are key determinants of prolonged hospitalization, increased healthcare costs, and elevated mortality risk (4). Accumulating evidence indicates that patients who develop PPCs are at increased risk for reduced long-term survival and poorer quality of life. Specifically, respiratory infections during follow-up have been identified as a risk factor for late mortality comparable to cancer recurrence after pneumonectomy, and patients who experience acute postoperative cardiopulmonary complications had a significantly higher incidence of cardiovascular or respiratory events in the chronic phase (5, 6). Given their prognostic significance, PPCs encompass a broad spectrum of respiratory abnormalities. In this study, the definition of PPCs is based on the European Perioperative Clinical Outcome (EPCO) definitions, including respiratory infections, respiratory failure, pleural effusion, atelectasis, pneumothorax, bronchospasm, and aspiration pneumonia (7).

Postoperative complications following lobectomy are closely associated with the overall health status of patients. Therefore, accurately predicting which patients are at heightened risk for these complications is a critical goal of perioperative care. Currently, common perioperative risk assessment tools in clinical practice include the American Society of Anesthesiologists (ASA) score, the Acute Physiology and Chronic Health Evaluation (APACHE/APACHE II) score, Body Mass Index (BMI), age, and the National Nosocomial Infections Surveillance (NNIS) risk index (8–10). However, these tools have clear limitations. For example, the ASA classification is inherently subjective and fails to adequately capture the compensatory capacity of multiple organ systems (11, 12). Similarly, single predictors like age or BMI do not reflect the complex, multisystem physiological decline that characterizes frailty (13, 14).

To address this gap, the concept of frailty has gained increasing attention in perioperative medicine. In recent years, frailty, which encompasses the decline of multiple physiological systems, has been widely recognized as an important clinical marker for predicting postoperative outcomes in elderly patients (14, 15). It refers to a non-specific condition where the body’s physiological reserve is reduced due to pathological states or the decline of various organs, making the patient’s ability to resist stressors weakened, and consequently, the body becomes more vulnerable to injury (16). Multiple validated frailty assessment tools exist, ranging from comprehensive geriatric assessments to simplified indices (14, 17–24). We selected the 5-factor modified frailty index (mFI-5) for this study because it better addresses the clinical demand for a more streamlined frailty measurement tool (25).

Although the relationship between frailty and postoperative complications has been widely studied, with a recent meta-analysis confirming the association between frailty and PPCs following pulmonary resection (25), several important knowledge gaps remain. First, the majority of existing studies have employed composite outcome measures, which may obscure differential associations between frailty and distinct types of PPCs, such as infection versus pneumothorax. Second, while simplified tools like the 5-factor modified frailty index (mFI-5) have been validated for predicting overall morbidity, the extent to which its predictive capacity varies across specific pulmonary complications—after comprehensive adjustment for demographic, clinical, and intraoperative confounders—remains unclear.

This study aims to investigate the differential association between the mFI-5 and specific PPCs (postoperative pulmonary infection, pneumothorax, and pleural effusion) in patients undergoing elective pulmonary lobectomy. By clarifying the specific nature of the frailty-PPCs association, we hope to provide clinicians with a more nuanced understanding of how this simple preoperative tool can inform perioperative risk stratification and guide targeted preventive strategies.

## Materials and methods

### Study design

This retrospective cohort study was conducted at the First Affiliated Hospital of Shantou University Medical College. We reviewed the electronic medical records of patients who underwent lung resection surgery between January 1, 2011, and December 30, 2024. In accordance with the Declaration of Helsinki and the relevant STROBE guidelines, this study was approved by the Ethics Committee of the First Affiliated Hospital of Shantou University Medical College (NO. B-2024-071). The requirement for informed consent was waived due to the retrospective nature of the analysis.

### Participants

Patients were included if they underwent elective lobectomy for lung cancer. The following exclusion criteria were applied: pulmonary bullae resection; wedge resection or segmentectomy; open lobectomy or conversion to thoracotomy; pulmonary biopsy; microwave ablation for pulmonary tumors; combined operations at other sites; postoperative pathology confirming non-lung cancer; and other pulmonary procedures such as laceration repair, abscess drainage or foreign body removal.

### Data collection

Data were extracted from the hospital’s electronic medical record system. Covariates included demographic characteristics (age, gender), clinical history (smoking, alcohol use, comorbidities), preoperative laboratory values [white blood cell (WBC) count, hemoglobin (HGB), platelet (PLT) count, albumin (ALB), fibrinogen (FIB), serum creatinine (Scr)], intraoperative factors [American Society of Anesthesiologists (ASA) classification, anesthesia induction method, anesthesia maintenance method, type of postoperative analgesia pump, surgical site, surgical procedure, duration of surgery, primary surgeon, total intraoperative fluid volume].

### Frailty measurement

The primary exposure was the 5-item Modified Frailty Index (mFI-5), a validated and simplified tool derived from the original 11-factor index of the American College of Surgeons National Surgical Quality Improvement Program (ACS NSQIP) database (26).

The mFI-5 consists of five non-overlapping clinical conditions: (1) congestive heart failure (CHF), documented diagnosis of heart failure in the medical record, or history of heart failure with current treatment (e.g., diuretics, ACE inhibitors); (2) diabetes mellitus, documented diagnosis of type 2 diabetes requiring pharmacological management (oral hypoglycemic agents or insulin) prior to surgery; (3) hypertension requiring medication, documented diagnosis of hypertension with current use of antihypertensive medications; (4) non-independent functional status, requiring assistance with activities of daily living such as bathing, eating, dressing, using the toilet, moving around, etc.; (5) chronic obstructive pulmonary disease (COPD), documented diagnosis of COPD, or use of bronchodilators or inhaled corticosteroids for chronic respiratory symptoms, confirmed by preoperative pulmonary function testing when available. Each clinical condition contributes 1 point to the total score. Patients were stratified into three groups based on the total mFI-5 score: Robust (score = 0), Pre-frail (score = 1), and Frail (score ≥ 2) (25).

### Outcome measurement

Primary outcomes were three specific PPCs, including postoperative pulmonary infection, postoperative pneumothorax, and postoperative pleural effusion, defined according to the European Perioperative Clinical Outcome (EPCO) criteria (7).

Postoperative pulmonary infection was diagnosed if antibiotic therapy was initiated due to suspected respiratory infection and at least one of the following: newly developed or altered sputum characteristics, new or changing pulmonary infiltrates, fever, or leukocyte count > 12 × 10^9^/L.

Postoperative pneumothorax was defined according to the EPCO criteria as the presence of gas in the pleural cavity without surrounding vascular structures on chest radiography. However, recognizing that in thoracic surgery pneumothorax may represent a radiographic sign rather than a clinical complication, we additionally reviewed medical records to identify clinically significant events. Specifically, pneumothorax was classified as a complication only if it required intervention (e.g., needle aspiration, chest tube reinsertion) or was associated with prolonged air leak (defined as air leak lasting > 5 days) or respiratory symptoms. Clinically insignificant residual air pockets detected incidentally on routine postoperative chest X-rays without symptoms or need for intervention were not classified as complications.

Postoperative pleural effusion was diagnosed when chest radiography demonstrated blunted costophrenic angles, loss of sharp delineation of the ipsilateral diaphragm in an upright position, evidence of adjacent anatomical structure displacement, or (in supine position) unilateral hazy opacity with preserved vascular markings.

### Statistical analysis

This study employed a retrospective cohort design to analyze and investigate the relevant data. Descriptive statistics are presented as mean ± standard deviation for normally distributed continuous variables, median (interquartile range) for skewed data, and frequency (%) for categorical variables. Group comparisons were performed using ANOVA, Kruskal–Wallis, or chi-square tests as appropriate.

The association between mFI-5 groups and each postoperative complication was assessed using logistic regression across three models: a crude unadjusted model; Model I adjusted for age and sex; and Model II adjusted for all potentially relevant variables. Results are reported as odds ratios (OR) with 95% confidence intervals (CI). Covariates for Model II were selected *a priori* based on clinical relevance and prior literature, supplemented by univariate screening (*P* < 0.20) to identify potential confounders. Multicollinearity among covariates was assessed using variance inflation factor (VIF), variables with VIF > 5 were excluded from the final model to ensure stability.

Subgroup analyses and interaction tests were conducted to examine effect modification by key demographic and clinical variables. A *p*-value < 0.05 was considered statistically significant. All analyses were performed using Empower Stats software^1^ (X&Y Solutions, Inc., Boston, Massachusetts).

## Results

### Study participants

**FIGURE 1:**
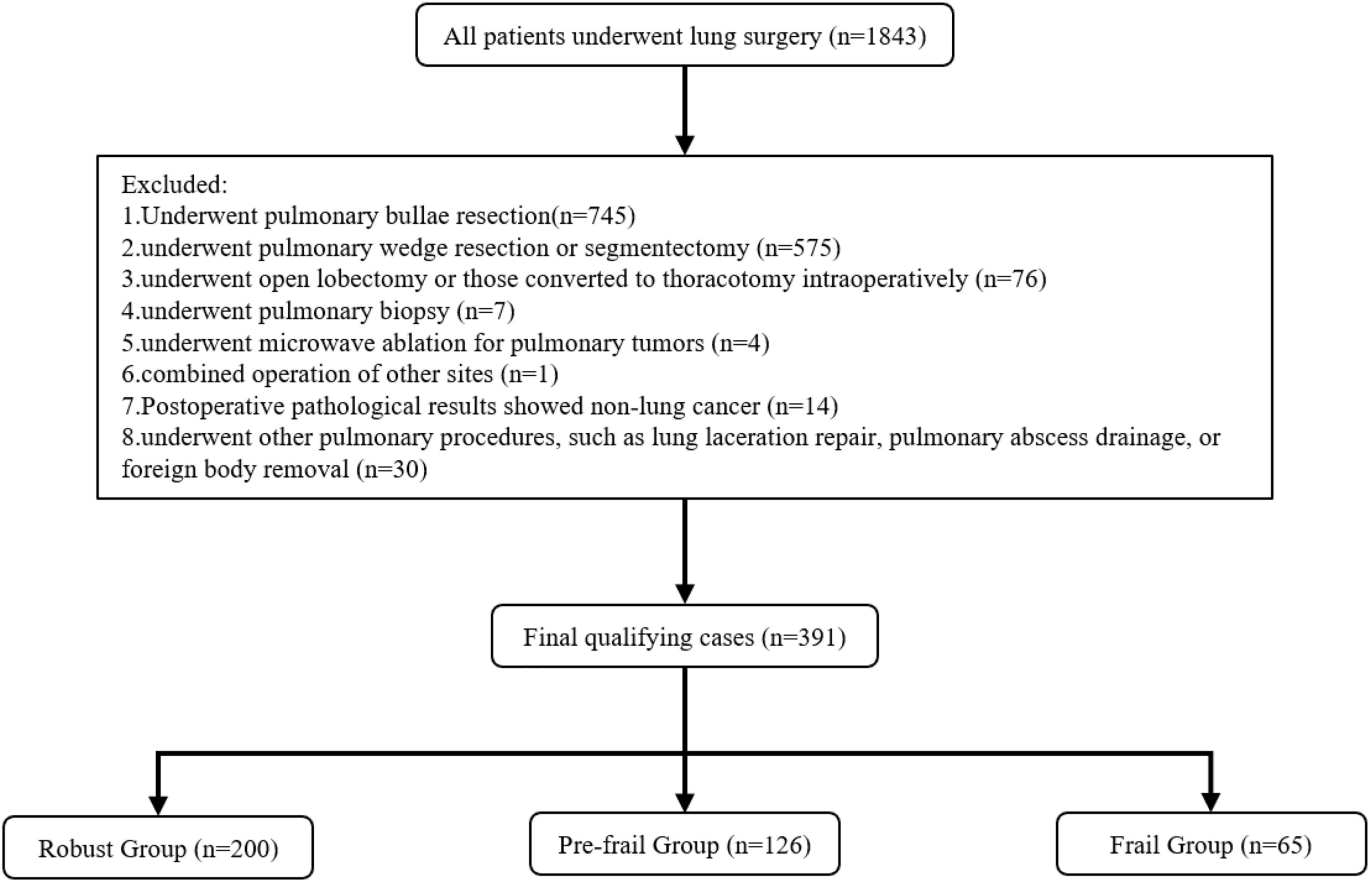
Flowchart of the process of patient enrollment.

The study initially involved a total of 1,843 participants. The entry time and selection deadline for participants were 2011-1-1 and 2024-12-30, respectively. Inclusion criteria included from January 01, 2011, to December 31, 2024, patients undergoing Lung surgery. Exclusion criteria included: (1) underwent pulmonary bullae resection (*n* = 745); (2) underwent pulmonary wedge resection or segmentectomy (*n* = 575); (3) underwent open lobectomy or those converted to thoracotomy intraoperatively (*n* = 76); (4) underwent pulmonary biopsy (*n* = 7); (5) underwent microwave ablation for pulmonary tumors (*n* = 4); (6) combined operation of other sites except esophagus (*n* = 1); (7) postoperative pathological results showed non-lung cancer (*n* = 14); (8) underwent other pulmonary procedures, such as lung laceration repair, pulmonary abscess drainage, or foreign body removal (*n* = 30). The final number of cases was 391 (Figure 1).

Table 1 displayed the clinical and demographic characteristics of the patients. Of the 391 patients, 239 were male (61.13%) and 152 were female (38.87%), with an average age of 62.17 ± 8.71 years. Regarding frailty, 200 (51.15%) were classified as robust, 126 (32.23%) were pre-frail, and 65 (16.62%) were frail.

**TABLE 1:** Baseline characteristics of participants (*N* = 391).

mFI-5	Robust group	Pre-frail group	Frail group	*P*-value
*N*	200	126	65	
General characteristics				
Age (years)	59.1 ± 9.1	64.6 ± 6.9	67.1 ± 6.9	< 0.001
Male	126 (63.0%)	72 (57.1%)	41 (63.1%)	0.538
Smoking history	90 (45.0%)	56 (44.4%)	26 (40.0%)	0.774
Alcohol history	23 (11.5%)	21 (16.7%)	9 (13.8%)	0.413
Preoperative comorbidities				
Hypertension	0 (0.0%)	71 (56.3%)	57 (87.7%)	< 0.001
Diabetes	0 (0.0%)	26 (20.6%)	35 (53.8%)	< 0.001
Neurological diseases	7 (3.5%)	21 (16.7%)	17 (26.2%)	< 0.001
History of cancer	24 (12.0%)	13 (10.3%)	4 (6.2%)	0.408
Preoperative laboratory tests				
WBC(10^9^/L)	6.7 ± 2.1	6.7 ± 1.8	7.6 ± 2.3	0.006
HGB (g/L)	129.9 ± 14.5	131.0 ± 15.9	124.8 ± 19.0	0.031
PLT (× 10^9^/L)	247.4 ± 82.0	234.4 ± 62.2	252.9 ± 84.6	0.200
ALB (g/L)	41.1 ± 5.2	41.7 ± 5.7	40.2 ± 7.7	0.006
SCr (μmol/L)	81.9 ± 16.6	81.6 ± 19.6	91.3 ± 30.9	0.003
FIB (g/L)	3.2 ± 0.9	3.2 ± 0.7	3.7 ± 1.3	< 0.001
Intraoperative related factors				
ASA III	37 (18.5%)	47 (37.3%)	41 (63.1%)	< 0.001
Left lung lobe surgery	88 (44.0%)	44 (34.9%)	27 (41.5%)	0.264
Thoracoscopic approach	173 (86.5%)	105 (83.3%)	54 (83.1%)	0.667
Surgery duration (min)	180.3 ± 53.6	179.9 ± 59.6	178.4 ± 51.1	0.884
Total intraoperative fluid volume (ml)	1552.5 ± 701.3	1467.1 ± 584.7	1457.6 ± 663.0	0.394
GEA	20 (10.0%)	10 (7.9%)	8 (12.3%)	0.615
TIVA	176 (88.0%)	110 (87.3%)	58 (89.2%)	0.927
PCIA	178 (90.4%)	118 (94.4%)	59 (93.7%)	0.375
Surgeon				
Surgeon A	90 (45.0%)	45 (35.7%)	23 (35.4%)	0.298
Surgeon B	16 (8.0%)	10 (7.9%)	2 (3.1%)	
Surgeon C	78 (39.0%)	55 (43.7%)	32 (49.2%)	
Surgeon D	16 (8.0%)	16 (12.7%)	8 (12.3%)	
PPCs				
Pulmonary Infection	52(26.0%)	43(34.1%)	23(35.4%)	0.151
Pneumothorax	60(30.0%)	35(27.8%)	11(16.9%)	0.137
Pleural Effusion	145(72.5%)	85(67.5%)	44(67.7%)	0.700

Data are expressed as Mean ± SD/Median (Q1–Q3)/*N* (%). PLT, platelet; SCr, serum creatinine; FIB, fibrinogen; ASA, American Society of Anesthesiologist Physical Status; GEA, general anesthesia combined with epidural; TIVA, total intravenous anesthesia; PCIA, patient controlled intravenous analgesia.

Significant differences were observed across groups for several variables. Participants in the Frail group were older (67.1 ± 6.9 years) compared to the Pre-frail (64.6 ± 6.9 years) and Robust groups (59.1 ± 9.1 years) (*P* < 0.001). The prevalence of preoperative comorbidities was markedly higher with increasing frailty: hypertension was present in 0.0, 56.3, and 87.7% of the Robust, Pre-frail, and Frail groups, respectively (*P* < 0.001); corresponding rates for diabetes were 0.0, 20.6, and 53.8% (*P* < 0.001); and for neurological diseases 3.5, 16.7, and 26.2% (*P* < 0.001). Preoperative laboratory tests also showed significant intergroup differences: the Frail group had higher white blood cell count (7.6 ± 2.3 × 10^9^/L, *P* = 0.006), lower hemoglobin (124.8 ± 19.0 g/L, *P* = 0.031), lower albumin (40.2 ± 7.7 g/L, *P* = 0.006), higher serum creatinine (91.3 ± 30.9 μmol/L, *P* = 0.003), and higher fibrinogen (3.7 ± 1.3 g/L, *P* < 0.001) compared to the other groups. The proportion of patients with ASA III physical status increased significantly across the frailty spectrum (18.5, 37.3, and 63.1%, *P* < 0.001).

In contrast, no statistically significant differences were found among groups for sex distribution, smoking or alcohol history, history of cancer, platelet count, laterality of lung lobe surgery, surgical approach (thoracoscopic vs. open), surgery duration, total intraoperative fluid volume, type of anesthesia (GEA or TIVA), use of postoperative PCIA, or distribution of surgeons (*P* > 0.05) (Table 1).

### Univariate analysis

Table 2 presents the results of univariate analysis for postoperative pulmonary complications. Univariate logistic regression models were used to perform regression analysis on postoperative pulmonary complications and their potential influencing factors, in order to evaluate the association between each variable and postoperative complications.

**TABLE 2:** Univariate analysis for PPCs.

Variables	Statistics	Postoperative pulmonary infection	Postoperative pneumothorax	Postoperative pleural effusion
		OR (95% CI)/*P*-valu	OR (95% CI)/*P*-value	OR (95% CI)/*P*-value
General characteristics				
Age (years)	62.2 ± 8.7	1.0 (1.0, 1.0) 0.743	1.0 (1.0, 1.0) 0.102	1.0 (1.0, 1.0) 0.271
≥60 (years)	256 (65.5%)	1.0 (0.6, 1.6) 0.945	0.9 (0.6, 1.4) 0.615	1.2 (0.8, 1.9) 0.374
Male	239 (61.1%)	1.0 (0.6, 1.5) 0.947	2.3 (1.4, 3.9) < 0.001	0.9 (0.6, 1.4) 0.587
Smoking history	172 (44.0%)	0.8 (0.5, 1.2) 0.212	1.8 (1.2, 2.9) 0.010	0.9 (0.6, 1.4) 0.770
Alcohol history	53 (13.6%)	1.7 (0.9, 3.1) 0.082	2.2 (1.2, 4.0) 0.009	1.0 (0.5, 1.8) 0.883
mFI-5				
Robust group	200 (51.2%)	Reference	Reference	Reference
Pre-frail group	126 (32.2%)	1.6 (1.0, 2.6) 0.061	0.9 (0.6, 1.5) 0.812	0.8 (0.5, 1.4) 0.466
Frail group	65 (16.6%)	1.7 (0.9, 3.1) 0.088	0.5 (0.2, 1.0) 0.057	0.9 (0.5, 1.6) 0.669
Preoperative comorbidities				
Hypertension	128 (32.7%)	1.1 (0.7, 1.8) 0.578	0.7 (0.4, 1.1) 0.141	0.8 (0.5, 1.3) 0.477
Diabetes	61 (15.6%)	1.1 (0.6, 2.0) 0.746	0.4 (0.2, 0.9) 0.026	0.7 (0.4, 1.3) 0.234
Neurological diseases	45 (11.5%)	1.1 (0.6, 2.1) 0.790	1.0 (0.5, 2.0) 0.991	1.1 (0.6, 2.2) 0.722
History of cancer	41 (10.5%)	0.8 (0.4, 1.6) 0.457	1.3 (0.7, 2.7) 0.435	0.7 (0.3, 1.3) 0.255
Preoperative laboratory tests				
WBC(10^9^/L)	6.8 ± 2.1	1.0 (0.9, 1.1) 0.916	1.0 (0.9, 1.1) 0.660	1.0 (0.9, 1.1) 0.336
HGB (g/L)	129.4 ± 15.8	1.0 (1.0, 1.0) 0.213	1.0 (1.0, 1.0) 0.651	1.0 (1.0, 1.0) 0.695
Anemia	145 (37.1%)	1.1 (0.7, 1.7) 0.756	1.1 (0.7, 1.7) 0.734	0.7 (0.5, 1.2) 0.194
PLT (× 10^9^/L)	244.1 ± 76.8	1.0 (1.0, 1.0) 0.070	1.0 (1.0, 1.0) 0.433	1.0 (1.0, 1.0) 0.400
Abnormal PLT	79(20.2%)	0.8(0.5,1.5)0.537	1.7(1.0,2.9)0.048	0.7(0.4,1.2)0.238
ALB (g/L)	41.2 ± 5.9	1.0 (1.0, 1.1) 0.119	1.0 (0.9, 1.0) 0.454	1.0 (1.0, 1.0) 0.948
Hypoalbuminemia	37(9.5%)	0.9(0.4,1.9)0.738	1.2(0.6,2.5)0.651	2.1(0.9,4.8)0.096
SCr (μmol/L)	83.3 ± 20.8	1.0 (1.0, 1.0) 0.564	1.0 (1.0, 1.0) 0.032	1.0 (1.0, 1.0) 0.719
≤100	321 (82.1%)	Reference	Reference	Reference
> 100	70 (17.9%)	0.6 (0.3, 1.1) 0.108	1.2 (0.7, 2.2) 0.478	1.4 (0.8, 2.5) 0.275
FIB (g/L)	3.3 ± 0.9	1.2 (1.0, 1.5) 0.068	1.1 (0.8, 1.4) 0.566	1.3 (1.0, 1.7) 0.031
Anesthesia and surgical related factors				
ASA I–II	266 (68.0%)	Reference	Reference	Reference
ASA III	125 (32.0%)	1.3 (0.8, 2.1) 0.213	0.9 (0.5, 1.4) 0.581	1.0 (0.6, 1.6) 1.000
Left lung lobe	159 (40.7%)	Reference	Reference	Reference
Right lung lobe	232 (59.3%)	1.7 (1.1, 2.6) 0.028	1.4 (0.9, 2.3) 0.144	0.6 (0.4, 1.0) 0.033
Thoracoscopic	332 (84.9%)	Reference	Reference	Reference
Single-port thoracoscopic	59 (15.1%)	1.7 (0.9, 3.0) 0.082	0.6 (0.3, 1.2) 0.137	0.7 (0.4, 1.2) 0.163
GEA	38 (9.7%)	Reference	Reference	Reference
GA	353 (90.3%)	2.0 (0.8, 4.6) 0.123	0.7 (0.3, 1.4) 0.266	0.5 (0.2, 1.1) 0.079
CIVIA	47 (12.0%)	Reference	Reference	Reference
TIVA	344 (88.0%)	0.7 (0.4, 1.3) 0.280	0.9 (0.5, 1.9) 0.861	1.0 (0.5, 2.0) 0.910
Surgery duration (min)	179.8 ± 55.1	1.0 (1.0, 1.0) 0.036	1.0 (1.0, 1.0) < 0.001	1.0 (1.0, 1.0) 0.472
≥180	182 (46.5%)	Reference	Reference	Reference
< 180	209 (53.5%)	0.5 (0.3, 0.8) 0.006	0.5 (0.3, 0.9) 0.008	1.1 (0.7, 1.7) 0.628
Total intraoperative fluid volume (mL)	1509.3 ± 659.3	1.0 (1.0, 1.0) 0.966	1.0 (1.0, 1.0) 0.140	1.0 (1.0, 1.0) 0.091
PCEA	30 (7.8%)	Reference	Reference	Reference
PCIA	355 (92.2%)	6.4 (1.5, 27.2) 0.012	0.8 (0.4, 1.9) 0.651	0.5 (0.2, 1.3) 0.167
Surgeon A	158 (40.4%)	Reference	Reference	Reference
Surgeon B	28 (7.2%)	1.1 (0.4, 2.7) 0.914	1.2 (0.5, 2.7) 0.746	1.1 (0.4, 3.0) 0.818
Surgeon C	165 (42.2%)	1.3 (0.8, 2.1) 0.366	0.7 (0.4, 1.2) 0.170	0.5 (0.3, 0.8) 0.006
Surgeon D	40 (10.2%)	4.3 (2.1, 8.8) < 0.001	1.0 (0.5, 2.2) 0.912	0.4 (0.2, 0.9) 0.017

Data are expressed as Mean ± SD/Median (Q1–Q3)/*N* (%). Abnormal PLT, platelet. > 300 or < 100 (10^9^/L); SCr, Serum creatinine; FIB, fibrinogen; ASA, American Society of Anesthesiologist Physical Status; GEA, general anesthesia combined with epidural; GA, general anesthesia; TIVA, total intravenous anesthesia; CIVIA, combined intravenous and inhalation anesthesia; PCEA, patient controlled epidural analgesia; PCIA, patient controlled intravenous analgesia; Hypoproteinemia, Albumin < 35 (g/L); Anemia, hemoglobin < 130 (g/L) in male or hemoglobin < 120 (g/L) in female.

For postoperative pulmonary infection, factors such as surgery duration, right lung lobe surgery, use of intravenous patient-controlled analgesia (IV PCA), and surgery performed by Surgeon D were found to be associated with postoperative pulmonary infection (*P* < 0.05). However, age, gender, alcohol history, smoking history, 5-factor modified frailty index, and preoperative histories of diabetes and hypertension showed no significant association. Preoperative neurological disease history, cancer history, and preoperative laboratory tests (WBC, HGB, PLT, ALB, SCR, FIB), ASA classification, surgical approach, anesthesia induction method, anesthesia maintenance method, and total intraoperative fluid volume had no significant effects on postoperative pulmonary infection (*P* > 0.05).

For postoperative pneumothorax, factors such as gender, smoking history, alcohol history, preoperative diabetes history, preoperative abnormal platelet count (platelets > 300 or < 100 (× 10^9^/L)), preoperative serum creatinine, and surgery duration were found to be significantly associated with postoperative pneumothorax (*P* < 0.05). In contrast, age, 5-factor modified frailty index, preoperative hypertension, neurological disease history, cancer history, preoperative WBC count, hemoglobin, platelet count, albumin, creatinine, fibrinogen, ASA classification, surgical site, surgical approach, anesthesia induction method, anesthesia maintenance method, and surgeon had no significant association with the occurrence of postoperative pneumothorax (*P* > 0.05).

For postoperative pleural effusion, preoperative fibrinogen, right lung lobe surgery, and surgery performed by Surgeons C and D were significantly associated with the risk of pleural effusion (*P* < 0.05). However, age, gender, smoking history, alcohol history, 5-factor modified frailty index, preoperative hypertension history, diabetes history, neurological disease history, cancer history, preoperative laboratory tests (WBC, HGB, PLT, ALB, SCR, FIB), ASA classification, surgical approach, anesthesia induction method, anesthesia maintenance method, surgery duration, and total intraoperative fluid volume showed no significant effect on postoperative pulmonary infection (*P* > 0.05).

### Results of unadjusted and adjusted logistic regression

In this study, three models were constructed to assess the independent effect of the 5-factor modified frailty index on postoperative complications using both univariate and multivariate logistic regression analyses. Table 3 presents the results of logistic regression analyses examining the association between the Modified Frailty Index (mFI-5) and the risk of postoperative pulmonary infection. In the unadjusted crude model, both the Pre-frail group (OR = 1.6, 95% CI: 1.0–2.6; *P* = 0.061) and the Frail group (OR = 1.7, 95% CI: 0.9–3.1; *P* = 0.088) showed a trend toward increased odds of infection compared to the Robust reference group, although these associations did not reach statistical significance. After adjustment for age and gender in Model I, the associations became statistically significant, with the Pre-frail group having 1.8 times the odds (95%CI: 1.1–3.0; *P* = 0.031) and the Frail group having 2.0 times the odds (95% CI: 1.0–3.7; *P* = 0.042). In the fully adjusted Model II, which controlled all covariates listed in Table 1, the strength of the associations increased substantially. The Pre-frail group had an odds ratio of 3.5 (95% CI: 1.6–7.9; *P* = 0.002), and the Frail group had an odds ratio of 7.3 (95% CI: 2.0–26.2; *P* = 0.002).

**TABLE 3:** Relationship between 5-factor modified frailty index and postoperative pulmonary infection.

	Crude model		Model 1		Model II	
mFI-5	OR (95% CI)	*P*-value	OR (95% CI)	*P*-value	OR (95% CI)	*P*-value
Robust group	Reference		Reference		Reference	
Pre-frail group	1.6 (1.0, 2.6)	0.061	1.8 (1.1, 3.0)	0.031	3.5 (1.6, 7.9)	0.002
Frail group	1.7 (0.9, 3.1)	0.088	2.0 (1.0, 3.7)	0.042	7.3 (2.0, 26.2)	0.002

The Crude model was unadjusted for any factors. Model I adjusted for age (years) and gender. Model II adjusted for age (years), gender, hypertension, diabetes, neurological diseases, cancer history, smoking history, alcohol history, WBC, HGB, PLT, ALB, SCr, FIB, surgical site, surgical approach, ASA classification, anesthesia method, anesthesia maintenance, surgery duration, total intraoperative fluid volume, postoperative pain management, and the surgeon.

Tables 4, 5 respectively present the associations between the 5-factor modified frailty index (mFI-5) and the risks of postoperative pneumothorax and pleural effusion. In the fully adjusted analyses (Model II), no statistically significant associations were observed for either complication (*P* > 0.05).

**TABLE 4:** Relationship between 5-factor modified frailty index and postoperative pneumothorax.

	Crude model		Model 1		Model II	
mFI-5	OR (95% CI)	*P*-value	OR (95% CI)	*P*-value	OR (95% CI)	*P*-value
Robust group	Reference		Reference		Reference	
Pre-frail group	0.9 (0.6, 1.5)	0.812	1.1 (0.7, 1.9)	0.651	1.3 (0.6, 3.1)	0.515
Frail group	0.5 (0.2, 1.0)	0.057	0.6 (0.3, 1.2)	0.165	1.0 (0.3, 4.0)	0.945

The Crude model was unadjusted for any factors. Model I adjusted for age (years) and gender. Model II adjusted for age (years), gender, hypertension, diabetes, neurological diseases, cancer history, smoking history, alcohol history, WBC, HGB, PLT, ALB, SCr, FIB, surgical site, surgical approach, ASA classification, anesthesia method, anesthesia maintenance, surgery duration, total intraoperative fluid volume, postoperative pain management, and the surgeon.

**TABLE 5:** Relationship between 5-factor modified frailty index and postoperative pleural effusion.

	Crude model		Model 1		Model II	
mFI-5	OR (95% CI)	*P*-value	OR (95% CI)	*P*-value	OR (95% CI)	*P*-value
Robust group	Reference		Reference		Reference	
Pre-frail group	0.8 (0.5, 1.4)	0.466	0.7 (0.4, 1.2)	0.233	0.9 (0.4, 2.1)	0.859
Frail group	0.9 (0.5, 1.6)	0.669	0.7 (0.4, 1.4)	0.355	1.3 (0.3, 5.2)	0.665

The Crude model was unadjusted for any factors. Model I adjusted for age (years) and gender. Model II adjusted for age (years), gender, hypertension, diabetes, neurological diseases, cancer history, smoking history, alcohol history, WBC, HGB, PLT, ALB, SCr, FIB, surgical site, surgical approach, ASA classification, anesthesia method, anesthesia maintenance, surgery duration, total intraoperative fluid volume, postoperative pain management, and the surgeon.

### Model diagnostics and sensitivity analyses

To ensure the stability and reliability of our multivariable models, we conducted comprehensive diagnostic assessments. Variance Inflation Factor (VIF) values for all covariates in the three final models were all well below 5 (range: 1.07–2.22), indicating no significant multicollinearity. Furthermore, sensitivity analyses using reduced covariate models yielded consistent results with our main findings (Supplementary Table 1), supporting the robustness of our conclusions.

### Subgroup analysis

Supplementary Tables 2–4 present stratified analyses to evaluate potential effect modifications on the associations between the 5-factor modified frailty index and postoperative pulmonary complications. For postoperative pulmonary infection (Supplementary Table 2), the increased risk associated with pre-frailty and frailty was more pronounced in specific subgroups, notably among males and patients with a smoking history, although the tests for interaction were not statistically significant (*P* for interaction = 0.2403 and 0.1112, respectively). In contrast, for postoperative pneumothorax (Supplementary Table 3), a statistically significant interaction was observed with smoking history (*P* for interaction = 0.0152), suggesting that the association between frailty and pneumothorax risk may differ between smokers and non-smokers. Regarding postoperative pleural effusion (Supplementary Table 4), a significant interaction was identified with the surgical site (*P* for interaction = 0.0233), indicating that the relationship between frailty status and effusion risk may vary between surgeries performed on the left vs. the right lung lobe.

## Discussion

This study demonstrates a differential predictive capacity of the mFI-5: it is strongly associated with postoperative pulmonary infection—with risk escalating from pre-frail (adjusted OR 3.5) to frail status (adjusted OR 7.3)—but not with pneumothorax or pleural effusion. These findings suggest that the predictive capacity of mFI-5 is complication-specific rather than uniform across all postoperative pulmonary complications.

Our results align with the growing body of literature linking frailty to adverse postoperative outcomes. A recent systematic review and meta-analysis by Tang et al. reported that frailty was significantly associated with an increased risk of PPCs following pulmonary resection (OR = 2.96, 95% CI: 2.15–4.06) (27). However, this analysis aggregated diverse complications and could not differentiate between infection and procedural complications. Our study extends this work by demonstrating that the predictive power of mFI-5 is particularly pronounced for infectious complications—a finding that persisted after comprehensive adjustment.

The association between frailty and postoperative pulmonary infection may be mediated by impaired compensatory capacity. Frail patients exhibit diminished mucociliary clearance, weakened cough reflex, and reduced immune competence (28, 29). These impairments are often exacerbated by intraoperative single-lung ventilation and tracheal intubation, which promote secretion retention and atelectasis (30, 31). Additionally, frail patients frequently require higher postoperative opioid doses for adequate analgesia, which further suppress the cough reflex and cause dose-dependent respiratory depression (32, 33).

In contrast, the absence of association between frailty and pneumothorax or pleural effusion indicates that these complications relate more to the surgical technique and adherence to postoperative protocols rather than the frail state of the patient (34, 35). For example, the choice of drainage methods and the duration of tube placement significantly influence the incidence of such complications (36). This distinction is clinically important: preventing pneumothorax or effusion likely requires optimization of operative technique and postoperative care pathways rather than patient-level intervention. The significant interactions we observed—between frailty and smoking for pneumothorax risk, and between frailty and surgical site for pleural effusion—suggest that frailty’s impact may be modulated by exposure and anatomical factors, warranting further investigation in larger cohorts.

The mFI-5 offers practical advantages for preoperative risk stratification. By drawing on routinely documented comorbidities, it provides a simple, low-burden assessment of a patient’s underlying health status—information crucial for tailoring perioperative care (37). Given the strong association between frailty and postoperative pulmonary infection observed in our study, interventions aimed at mitigating frailty-related risk warrant consideration. Prehabilitation—a multimodal approach encompassing exercise training, nutritional optimization, and psychological support—has emerged as a promising strategy to enhance physiological reserve and improve postoperative outcomes in frail surgical patients (38, 39). Our findings support the potential utility of mFI-5 as a tool to identify patients who might benefit most from such targeted preoperative optimization programs (40). Early identification of high-risk patients could enable timely interventions, potentially reducing both clinical morbidity and the substantial economic burden associated with postoperative pulmonary infections (41, 42). Early identification of high-risk patients could enable targeted perioperative interventions, potentially reducing both clinical morbidity and financial burden.

This study has several limitations. First, its single-center, retrospective design may limit generalizability due to institutional-specific practices and patient demographics; multicenter validation is needed. Second, although we adjusted for numerous covariates, unmeasured confounders—such as detailed pulmonary function (e.g., FEV1) and intraoperative physiological parameters—may influence outcomes. Third, as a retrospective study, the reliance on electronic health records introduces potential information bias. Additionally, the retrospective design precludes the establishment of causality and only allows for the identification of associations, thereby limiting the in-depth exploration of the mechanisms by which the mFI-5 exerts its effects. Fourth, the retrospective definition of pleural effusion based on radiographic findings may have included clinically insignificant events. Although our analysis showed no association between frailty and effusion, we acknowledge that a true association with clinically significant effusion cannot be ruled out. Prospective studies with standardized, clinically meaningful outcome definitions are needed to validate our findings.

## Conclusion

This study demonstrates that mFI-5 is a strong and independent predictor of postoperative pulmonary infection following pulmonary lobectomy, with the risk escalating progressively from the pre-frail to the frail status. In contrast, frailty status was not significantly associated with postoperative pneumothorax or pleural effusion, indicating that the predictive capacity of mFI-5 is complication-specific. Future multicenter prospective studies are warranted to validate the clinical utility of the mFI-5 and to explore whether frailty-guided perioperative interventions can reduce infectious complications after lobectomy.

## Data Availability

The raw data supporting the conclusions of this article will be made available by the authors, without undue reservation.

## Footnotes

1
www.empowerstats.com
