# Correction: Differential association of the 5-factor modified frailty index with postoperative pulmonary complications: specific prediction of infection risk after pulmonary lobectomy

**DOI:** 10.3389/fmed.2026.1855349

**Published:** 2026-05-04

**Authors:** Qianying Zheng, Zhuangbo Wu, Lingbo Du, Aiyang Chen, Jingmei Zhou, Jingxin Shi, Yuqing Lai, Wenbo Chen, Weiqi Ke, Huali Xu

**Affiliations:** 1 Department of Anesthesiology, The First Affiliated Hospital of Shantou University Medical College, Shantou, Guangdong, China; 2 Department of Anesthesiology, Jieyang Ciyun Hospital, Jieyang, Guangdong, China; 3 Shantou University Medical College, Shantou, Guangdong, China; 4 Department of Anesthesiology, Jieyang Haoze Hospital, Jieyang, Guangdong, China; 5 Department of Anesthesiology, People's Hospital of Shantou, Shantou, Guangdong, China; 6 Department of Anesthesiology, Nanfang Hospital, Southern Medical University, Guangzhou, Guangdong, China

**Keywords:** frailty, lobectomy, modified frailty index, pleural effusion, pneumothorax, postoperative infection

There was a mistake in Table 2 as published.

**Table 2:** Univariate analysis for PPCs.

Variables	Statistics	Postoperative pulmonary infection	Postoperative pneumothorax	Postoperative pleural effusion
		OR (95%CI)/*P*-value	OR (95%CI)/*P*-value	OR (95%CI)/*P*-value
General characteristics				
Age (years)	62.2 ± 8.7	1.0 (1.0, 1.0) 0.743	1.0 (1.0, 1.0) 0.102	1.0 (1.0, 1.0) 0.271
≥60 (years)	256 (65.5%)	1.0 (0.6, 1.6) 0.945	0.9 (0.6, 1.4) 0.615	1.2 (0.8, 1.9) 0.374
Male	239 (61.1%)	1.0 (0.6, 1.5) 0.947	2.3 (1.4, 3.9) < 0.001	0.9 (0.6, 1.4) 0.587
Smoking history	172 (44.0%)	0.8 (0.5, 1.2) 0.212	1.8 (1.2, 2.9) 0.010	0.9 (0.6, 1.4) 0.770
Alcohol history	53 (13.6%)	1.7 (0.9, 3.1) 0.082	2.2 (1.2, 4.0) 0.009	1.0 (0.5, 1.8) 0.883
mFI-5				
Robust group	200 (51.2%)	Reference	Reference	Reference
Pre-frail group	126 (32.2%)	1.6 (1.0, 2.6) 0.061	0.9 (0.6, 1.5) 0.812	0.8 (0.5, 1.4) 0.466
Frail group	65 (16.6%)	1.7 (0.9, 3.1) 0.088	0.5 (0.2, 1.0) 0.057	0.9 (0.5, 1.6) 0.669
Preoperative comorbidities				
Hypertension	128 (32.7%)	1.1 (0.7, 1.8) 0.578	0.7 (0.4, 1.1) 0.141	0.8 (0.5, 1.3) 0.477
Diabetes	61 (15.6%)	1.1 (0.6, 2.0) 0.746	0.4 (0.2, 0.9) 0.026	0.7 (0.4, 1.3) 0.234
Neurological diseases	45 (11.5%)	1.1 (0.6, 2.1) 0.790	1.0 (0.5, 2.0) 0.991	1.1 (0.6, 2.2) 0.722
History of cancer	41 (10.5%)	0.8 (0.4, 1.6) 0.457	1.3 (0.7, 2.7) 0.435	0.7 (0.3, 1.3) 0.255
Preoperative laboratory tests				
WBC (10^9^/L)	6.8 ± 2.1	1.0 (0.9, 1.1) 0.916	1.0 (0.9, 1.1) 0.660	1.0 (0.9, 1.1) 0.336
HGB (g/L)	129.4 ± 15.8	1.0 (1.0, 1.0) 0.213	1.0 (1.0, 1.0) 0.651	1.0 (1.0, 1.0) 0.695
Anemia	145 (37.1%)	1.1 (0.7, 1.7) 0.756	1.1 (0.7, 1.7) 0.734	0.7 (0.5, 1.2) 0.194
PLT ( × 10^9^/L)	244.1 ± 76.8	1.0 (1.0, 1.0) 0.070	1.0 (1.0, 1.0) 0.433	1.0 (1.0, 1.0) 0.400
Abnormal PLT	79 (20.2%)	0.8 (0.5, 1.5)0.537	1.7 (1.0, 2.9)0.048	0.7 (0.4, 1.2)0.238
ALB (g/L)	41.2 ± 5.9	1.0 (1.0, 1.1) 0.119	1.0 (0.9, 1.0) 0.454	1.0 (1.0, 1.0) 0.948
Hypoalbuminemia	37 (9.5%)	0.9 (0.4, 1.9)0.738	1.2 (0.6, 2.5)0.651	2.1 (0.9, 4.8)0.096
SCr (μmol/L)	83.3 ± 20.8	1.0 (1.0, 1.0) 0.564	1.0 (1.0, 1.0) 0.032	1.0 (1.0, 1.0) 0.719
≤ 100	321 (82.1%)	Reference	Reference	Reference
>100	70 (17.9%)	0.6 (0.3, 1.1) 0.108	1.2 (0.7, 2.2) 0.478	1.4 (0.8, 2.5) 0.275
FIB (g/L)	3.3 ± 0.9	1.2 (1.0, 1.5) 0.068	1.1 (0.8, 1.4) 0.566	1.3 (1.0, 1.7) 0.031
Anesthesia and surgical related factors				
ASA I–II	266 (68.0%)	Reference	Reference	Reference
ASA III	125 (32.0%)	1.3 (0.8, 2.1) 0.213	0.9 (0.5, 1.4) 0.581	1.0 (0.6, 1.6) 1.000
Left lung lobe	159 (40.7%)	Reference	Reference	Reference
Right lung lobe	232 (59.3%)	1.7 (1.1, 2.6) 0.028	1.4 (0.9, 2.3) 0.144	0.6 (0.4, 1.0) 0.033
Thoracoscopic	332 (84.9%)	Reference	Reference	Reference
Single-port thoracoscopic	59 (15.1%)	1.7 (0.9, 3.0) 0.082	0.6 (0.3, 1.2) 0.137	0.7 (0.4, 1.2) 0.163
GEA	38 (9.7%)	Reference	Reference	Reference
GA	353 (90.3%)	2.0 (0.8, 4.6) 0.123	0.7 (0.3, 1.4) 0.266	0.5 (0.2, 1.1) 0.079
CIVIA	47 (12.0%)	Reference	Reference	Reference
TIVA	344 (88.0%)	0.7 (0.4, 1.3) 0.280	0.9 (0.5, 1.9) 0.861	1.0 (0.5, 2.0) 0.910
Surgery duration (min)	179.8 ± 55.1	1.0 (1.0, 1.0) 0.036	1.0 (1.0, 1.0) < 0.001	1.0 (1.0, 1.0) 0.472
≥180	182 (46.5%)	Reference	Reference	Reference
< 180	209 (53.5%)	0.5 (0.3, 0.8) 0.006	0.5 (0.3, 0.9) 0.008	1.1 (0.7, 1.7) 0.628
Total intraoperative fluid volume (ml)	1509.3 ± 659.3	1.0 (1.0, 1.0) 0.966	1.0 (1.0, 1.0) 0.140	1.0 (1.0, 1.0) 0.091
PCEA	30 (7.8%)	Reference	Reference	Reference
PCIA	355 (92.2%)	6.4 (1.5, 27.2) 0.012	0.8 (0.4, 1.9) 0.651	0.5 (0.2, 1.3) 0.167
Surgeon A	158 (40.4%)	Reference	Reference	Reference
Surgeon B	28 (7.2%)	1.1 (0.4, 2.7) 0.914	1.2 (0.5, 2.7) 0.746	1.1 (0.4, 3.0) 0.818
Surgeon C	165 (42.2%)	1.3 (0.8, 2.1) 0.366	0.7 (0.4, 1.2) 0.170	0.5 (0.3, 0.8) 0.006
Surgeon D	40 (10.2%)	4.3 (2.1, 8.8) < 0.001	1.0 (0.5, 2.2) 0.912	0.4 (0.2, 0.9) 0.017

Data are expressed as mean ± SD/median (Q1–Q3)/*N* (%). Abnormal PLT, platelet. >300 or < 100 (10^9^/L); SCr, Serum creatinine; FIB, fibrinogen; ASA, American Society of Anesthesiologist Physical Status; GEA, general anesthesia combined with epidural; GA, general anesthesia; TIVA, total intravenous anesthesia; CIVIA, combined intravenous and inhalation anesthesia; PCEA, patient controlled epidural analgesia; PCIA, patient controlled intravenous analgesia; Hypoproteinemia, Albumin < 35 (g/L); Anemia, hemoglobin < 130 (g/L) in male or hemoglobin < 120 (g/L) in female.

Error 1—Column header misalignment.

The three complication column headers—“Postoperative pulmonary infection”, “Postoperative pneumothorax”, and “Postoperative pleural effusion”—were not correctly aligned with their corresponding OR (95% CI) and *P*-value columns. As a result, the odds ratios and *P*-values shown under each complication header did not belong to that complication.

Error 2—Incorrect use of “=” instead of “≥” or “ ≤ ” (three places)

In the Variables column, the symbol “=” was incorrectly used in three grouping indicators: “=60 (years)” should be “≥60 (years)”, “=100” (under SCr) should be “ ≤ 100”, “=180” (under Surgery duration) should be “≥180”. No other data or statistical results are affected.

The corrected Table 2 appears below.

The original version of this article has been updated.

