# Supplementary material for: Differential association of the 5-factor modified frailty index with postoperative pulmonary complications: specific prediction of infection risk after pulmonary lobectomy
Source: Front Med (Lausanne). 2026 Apr 13;13:1771113. doi: 10.3389/fmed.2026.1771113 (PMC13110951; doi:10.3389/fmed.2026.1771113)
Supplement: Supplementary file 1 [file Table_1.DOCX]

**Table S1 Relationship between 5-Factor Modified Frailty Index and PPCs**

|  | **Postoperative Pulmonary Infection** | | **Postoperative Pneumothorax** | | **Postoperative Pleural Effusion** | |
| --- | --- | --- | --- | --- | --- | --- |
| **mFI-5** | **OR (95%CI)** | **P -value** | **OR (95%CI)** | **P -value** | **OR (95%CI)** | **P -value** |
| Robust group | Reference | | Reference | | Reference | |
| Pre-frail group | 2.7 (1.3, 5.7) | 0.007 | 1.5 (0.7, 3.2) | 0.351 | 1.0 (0.5, 2.2) | 0.963 |
| Frail group | 4.6 (1.5, 14.2) | 0.007 | 1.2 (0.3, 4.0) | 0.795 | 1.4 (0.4, 4.9) | 0.556 |

Adjusted for age (years), gender, hypertension, diabetes, smoking history, alcohol history, surgical site, ASA classification, surgery duration.

**Table S2 Stratified Analysis of the 5-Factor Modified Frailty Index and Postoperative Pulmonary Infection**

|  |  | **Robust group** | **Pre-frail group** | **Frail group** |  |
| --- | --- | --- | --- | --- | --- |
|  | **N** |  | **β (95%CI)** | **β (95%CI)** | **P for interaction** |
| General Characteristics |  |  |  |  |  |
| Age (years) |  |  |  |  | 0.8062 |
| <60 | 135 | Reference | 2.0 (0.9, 4.8) | 1.4 (0.3, 6.2) |  |
| >=60 | 256 | Reference | 1.5 (0.8, 2.9) | 1.8 (0.9, 3.7) |  |
| Gender |  |  |  |  | 0.2403 |
| Female | 152 | Reference | 0.7 (0.3, 1.6) | 0.9 (0.3, 2.3) |  |
| Male | 239 | Reference | 2.7 (1.4, 5.2) | 2.6 (1.2, 5.6) |  |
| Smoking history |  |  |  |  | 0.1112 |
| no | 219 | Reference | 1.1 (0.6, 2.1) | 1.1 (0.5, 2.4) |  |
| yes | 172 | Reference | 2.8 (1.3, 6.1) | 3.1 (1.2, 8.2) |  |
| Alcohol history |  |  |  |  | 0.4904 |
| no | 338 | Reference | 1.4 (0.8, 2.3) | 1.6 (0.8, 3.1) |  |
| yes | 53 | Reference | 3.1 (0.9, 11.0) | 2.3 (0.5, 11.3) |  |
| Preoperative Comorbidities | |  |  |  |  |
| Neurological diseases |  |  |  |  | 0.8334 |
| no | 346 | Reference | 1.6 (0.9, 2.6) | 1.7 (0.8, 3.3) |  |
| yes | 45 | Reference | 3.0 (0.3, 30.0) | 3.3 (0.3, 33.9) |  |
| History of cancer |  |  |  |  | 0.6416 |
| no | 350 | Reference | 1.5 (0.9, 2.5) | 1.6 (0.9, 3.1) |  |
| yes | 41 | Reference | 3.1 (0.7, 14.7) | 1.7 (0.1, 20.4) |  |
| Preoperative Laboratory Tests | | |  |  |  |
| WBC（10^9^/L) |  |  |  |  | 0.6529 |
| >10 | 28 | Reference | 3.0 (0.2, 59.9) | 5.1 (0.4, 59.5) |  |
| <=10 | 363 | Reference | 1.5 (0.9, 2.5) | 1.7 (0.9, 3.1) |  |
| Anemia |  |  |  |  | 0.3080 |
| no | 246 | Reference | 1.3 (0.7, 2.3) | 1.2 (0.5, 2.8) |  |
| yes | 145 | Reference | 2.5 (1.1, 5.9) | 2.6 (1.1, 6.4) |  |
| PLT(×10^9^/L) |  |  |  |  | 0.5746 |
| Normal PLT | 312 | Reference | 1.5 (0.9, 2.5) | 1.9 (0.9, 3.8) |  |
| Abnormal PLT | 79 | Reference | 2.3 (0.7,7.8) | 1.3 (0.4,4.6) |  |
| ALB(g/L） |  |  |  |  | 0.7122 |
| <35 | 37 | Reference | 0.9 (0.1, 5.8) | 1.0 (0.2, 5.2) |  |
| >=35 | 354 | Reference | 1.7 (1.0, 2.8) | 1.8 (1.0, 3.5) |  |
| SCr(μmol/L) |  |  |  |  | 0.5489 |
| <=100 | 321 | Reference | 1.5 (0.9, 2.5) | 1.5 (0.8, 2.9) |  |
| >100 | 70 | Reference | 2.3 (0.6, 9.2) | 3.6 (0.8, 15.1) |  |
| FIB(g/L) |  |  |  |  | 0.8152 |
| <=4 | 321 | Reference | 1.5 (0.9, 2.6) | 1.5 (0.7, 3.2) |  |
| >4 | 65 | Reference | 2.4 (0.7, 9.1) | 1.9 (0.6, 6.2) |  |
| Anesthesia and Surgical Related Factors | | |  |  |  |
| ASA |  |  |  |  | 0.8005 |
| I -II | 266 | Reference | 1.7 (0.9, 3.0) | 1.3 (0.5, 3.4) |  |
| III | 125 | Reference | 1.4 (0.5, 3.6) | 1.7 (0.7, 4.5) |  |
| Surgical Site |  |  |  |  | 0.8877 |
| Left lung lobe | 159 | Reference | 1.3 (0.6, 3.1) | 1.6 (0.6, 4.3) |  |
| Right lung lobe | 232 | Reference | 1.7 (0.9, 3.1) | 1.7 (0.8, 3.7) |  |
| Thoracoscopic approach |  |  |  |  | 0.9951 |
| Thoracoscopy | 332 | Reference | 1.6 (0.9, 2.7) | 1.7 (0.9, 3.2) |  |
| Single-port thoracoscopic | 59 | Reference | 1.5 (0.5, 4.9) | 1.7 (0.4, 7.0) |  |
| Anesthesia Method |  |  |  |  | 0.9824 |
| GEA | 38 | Reference | 1.4 (0.2, 10.2) | 1.9 (0.3, 14.2) |  |
| GA | 353 | Reference | 1.6 (1.0, 2.6) | 1.7 (0.9, 3.2) |  |
| Anesthesia Maintenance |  |  |  |  | 0.6624 |
| CIVIA | 47 | Reference | 1.6 (0.4, 5.7) | 0.8 (0.1, 5.1) |  |
| TIVA | 344 | Reference | 1.6 (0.9, 2.7) | 1.9 (1.0, 3.5) |  |
| Surgery duration（min） |  |  |  |  | 0.4833 |
| >=180 | 182 | Reference | 1.8 (0.9, 3.5) | 2.5 (1.1, 5.7) |  |
| <180 | 209 | Reference | 1.5 (0.8, 3.1) | 1.2 (0.5, 2.9) |  |
| Total intraoperative fluid volume (ml) | | |  |  | 0.1713 |
| 100 - 1050 | 97 | Reference | 0.7 (0.3, 2.0) | 0.9 (0.3, 3.4) |  |
| 1100 - 1600 | 157 | Reference | 1.7 (0.8, 3.8) | 2.9 (1.2, 7.2) |  |
| 1700 - 4500 | 136 | Reference | 2.5 (1.1, 5.7) | 1.2 (0.4, 3.6) |  |
| Postoperative Pain Management Mode | | | |  | 0.3467 |
| PCEA | 30 | Reference | NA | 6.0 (0.3, 124.1) |  |
| PCIA | 355 | Reference | 1.6 (1.0, 2.7) | 1.7 (0.9, 3.2) |  |
| Surgeon |  |  |  |  | 0.8518 |
| Surgeon A | 158 | Reference | 2.3 (1.0, 5.3) | 2.0 (0.7, 5.7) |  |
| Surgeon B | 28 | Reference | 0.7 (0.1, 5.1) | 3.0 (0.2, 59.9) |  |
| Surgeon C | 165 | Reference | 1.1 (0.5, 2.4) | 1.2 (0.5, 3.0) |  |
| Surgeon D | 40 | Reference | 1.7 (0.4, 6.8) | 1.7 (0.3, 9.4) |  |

**Abbreviations:**

Abnormal PLT, platelet.>300or<100(10^9/L); SCr, Serum creatinine; FIB, fibrinogen; ASA, American Society of Anesthesiologist Physical Status; GEA, general anesthesia combined with epidural; GA, general anesthesia; TIVA, total intravenous anesthesia; CIVIA, combined intravenous-inhalation anesthesia; PCEA, patient controlled epidural analgesia; PCIA, patient controlled intravenous analgesia; Anemia, hemoglobin <130(g/L) in male or hemoglobin<120(g/L) in female.

**Table S3 Stratified Analysis of the 5-Factor Modified Frailty Index on Postoperative Pneumothorax**

|  |  | **Robust group** | **Pre-frail group** | **Frail group** |  |
| --- | --- | --- | --- | --- | --- |
|  | **N** |  | **β (95%CI)** | **β (95%CI)** | **P for interaction** |
| General Characteristics |  |  |  |  |  |
| Age (years) |  |  |  |  | 0.2504 |
| <60 | 135 | Reference | 0.6 (0.2, 1.7) | 1.2 (0.3, 5.0) |  |
| >=60 | 256 | Reference | 1.1 (0.6, 2.0) | 0.4 (0.2, 1.0) |  |
| Gender |  |  |  |  | 0.4896 |
| Female | 152 | Reference | 0.6 (0.2, 1.6) | 0.3 (0.1, 1.6) |  |
| Male | 239 | Reference | 1.2 (0.7, 2.2) | 0.6 (0.2, 1.3) |  |
| Smoking history |  |  |  |  | 0.0152 |
| no | 219 | Reference | 0.5 (0.2, 1.1) | 0.2 (0.1, 0.7) |  |
| yes | 172 | Reference | 1.7 (0.9, 3.5) | 1.1 (0.4, 2.8) |  |
| Alcohol history |  |  |  |  | 0.2453 |
| no | 338 | Reference | 0.8 (0.5, 1.4) | 0.4 (0.2, 0.9) |  |
| yes | 53 | Reference | 1.7 (0.5, 5.7) | 1.5 (0.3, 7.2) |  |
| Preoperative Comorbidities | |  |  |  |  |
| Neurological diseases |  |  |  |  | 0.3833 |
| no | 346 | Reference | 0.8 (0.5, 1.4) | 0.5 (0.2, 1.1) |  |
| yes | 45 | Reference | 3.7 (0.4, 36.6) | 1.3 (0.1, 15.0) |  |
| History of cancer |  |  |  |  | 0.3496 |
| no | 350 | Reference | 1.0 (0.6, 1.7) | 0.6 (0.3, 1.2) |  |
| yes | 41 | Reference | 0.7 (0.2, 3.1) | NA |  |
| Preoperative Laboratory Tests | | |  |  |  |
| WBC（10^9^/L) |  |  |  |  | 0.6529 |
| >10 | 28 | Reference | 3.0 (0.2, 59.9) | 5.1 (0.4, 59.5) |  |
| <=10 | 363 | Reference | 0.9 (0.6, 1.5) | 0.5 (0.2, 1.1) |  |
| Anemia |  |  |  |  | 0.7547 |
| no | 246 | Reference | 0.9 (0.5, 1.6) | 0.4 (0.1, 1.2) |  |
| yes | 145 | Reference | 1.1 (0.5, 2.6) | 0.6 (0.2, 1.6) |  |
| PLT(×10^9^/L) |  |  |  |  | 0.4452 |
| Normal PLT | 312 | Reference | 1.1 (0.7, 2.0) | 0.4 (0.2, 1.1) |  |
| Abnormal PLT | 79 | Reference | 0.5 (0.2,1.7) | 0.5 (0.1,1.6) |  |
| ALB(g/L） |  |  |  |  | 0.4790 |
| <35 | 37 | Reference | 2.6 (0.5, 14.6) | 0.6 (0.1, 3.7) |  |
| >=35 | 354 | Reference | 0.9 (0.5, 1.5) | 0.5 (0.2, 1.1) |  |
| SCr(μmol/L) |  |  |  |  | 0.1665 |
| <=100 | 321 | Reference | 0.8 (0.5, 1.4) | 0.4 (0.2, 0.9) |  |
| >100 | 70 | Reference | 2.3 (0.7, 7.4) | 1.2 (0.3, 5.0) |  |
| FIB(g/L) |  |  |  |  | 0.8152 |
| <=4 | 321 | Reference | 1.5 (0.9, 2.6) | 1.5 (0.7, 3.2) |  |
| >4 | 65 | Reference | 2.4 (0.7, 9.1) | 1.9 (0.6, 6.2) |  |
| Anesthesia and Surgical Related Factors | | |  |  |  |
| ASA |  |  |  |  | 0.0519 |
| I -II | 266 | Reference | 1.0 (0.5, 1.7) | 0.1 (0.0, 0.8) |  |
| III | 125 | Reference | 1.1 (0.4, 2.9) | 1.0 (0.4, 2.8) |  |
| Surgical Site |  |  |  |  | 0.3849 |
| Left lung lobe | 159 | Reference | 0.9 (0.4, 2.2) | 0.2 (0.0, 1.0) |  |
| Right lung lobe | 232 | Reference | 0.9 (0.5, 1.7) | 0.7 (0.3, 1.6) |  |
| Thoracoscopic approach | |  |  |  | 0.7532 |
| Thoracoscopy | 332 | Reference | 0.9 (0.6, 1.6) | 0.5 (0.2, 1.0) |  |
| Single-port thoracoscopic | 59 | Reference | 1.0 (0.2, 4.5) | 1.0 (0.2, 6.0) |  |
| Anesthesia Method |  |  |  |  | 0.1732 |
| GEA | 38 | Reference | 3.0 (0.6, 14.9) | 1.8 (0.3, 10.4) |  |
| GA | 353 | Reference | 0.8 (0.5, 1.4) | 0.4 (0.2, 0.9) |  |
| Anesthesia Maintenance |  |  |  |  | 0.9381 |
| CIVIA | 47 | Reference | 1.1 (0.3, 4.4) | 0.4 (0.0, 4.0) |  |
| TIVA | 344 | Reference | 0.9 (0.5, 1.6) | 0.5 (0.2, 1.1) |  |
| Surgery duration（min） |  |  |  |  | 0.8867 |
| >=180 | 182 | Reference | 1.1 (0.5, 2.1) | 0.5 (0.2, 1.2) |  |
| <180 | 209 | Reference | 0.9 (0.4, 1.8) | 0.5 (0.2, 1.6) |  |
| Total intraoperative fluid volume (ml) | | |  |  | 0.7369 |
| 100 - 1050 | 97 | Reference | 0.5 (0.2, 1.6) | 0.4 (0.1, 1.8) |  |
| 1100 - 1600 | 157 | Reference | 0.9 (0.4, 2.0) | 0.5 (0.2, 1.4) |  |
| 1700 - 4500 | 136 | Reference | 1.4 (0.6, 3.1) | 0.7 (0.2, 2.3) |  |
| Postoperative Pain Management Mode | | | |  | 0.3103 |
| PCEA | 30 | Reference | 1.1 (0.2, 7.7) | 2.8 (0.3, 25.5) |  |
| PCIA | 355 | Reference | 0.9 (0.5, 1.5) | 0.4 (0.2, 1.0) |  |
| Surgeon |  |  |  |  | 0.3532 |
| Surgeon A | 158 | Reference | 1.4 (0.6, 2.9) | 0.5 (0.2, 1.7) |  |
| Surgeon B | 28 | Reference | 3.0 (0.6, 16.1) | NA |  |
| Surgeon C | 165 | Reference | 0.7 (0.3, 1.6) | 0.5 (0.2, 1.5) |  |
| Surgeon D | 40 | Reference | 0.3 (0.1, 1.5) | 0.4 (0.1, 2.8) |  |

**Abbreviations:**

Abnormal PLT, platelet.>300or<100(10^9/L); SCr, Serum creatinine; FIB, fibrinogen; ASA, American Society of Anesthesiologist Physical Status; GEA, general anesthesia combined with epidural; GA, general anesthesia; TIVA, total intravenous anesthesia; CIVIA, combined intravenous-inhalation anesthesia; PCEA, patient controlled epidural analgesia; PCIA, patient controlled intravenous analgesia; Anemia, hemoglobin <130(g/L) in male or hemoglobin<120(g/L) in female.

**Table S4 Stratified Analysis of the 5-Factor Modified Frailty Index on Postoperative Pleural Effusion**

|  |  | **Robust group** | **Pre-frail group** | **Frail group** |  |
| --- | --- | --- | --- | --- | --- |
|  | **N** |  | **β (95%CI)** | **β (95%CI)** | **P for interaction** |
| General Characteristics |  |  |  |  |  |
| Age (years) |  |  |  |  | 0.9452 |
| <60 | 135 | Reference | 0.8 (0.3, 1.9) | 1.0 (0.2, 4.2) |  |
| >=60 | 256 | Reference | 0.8 (0.4, 1.4) | 0.8 (0.4, 1.5) |  |
| Gender |  |  |  |  | 0.0888 |
| Female | 152 | Reference | 0.7 (0.3, 1.4) | 2.0 (0.6, 6.5) |  |
| Male | 239 | Reference | 1.0 (0.5, 1.8) | 0.6 (0.3, 1.3) |  |
| Smoking history |  |  |  |  | 0.2514 |
| no | 219 | Reference | 1.0 (0.5, 1.9) | 1.4 (0.6, 3.1) |  |
| yes | 172 | Reference | 0.7 (0.3, 1.3) | 0.5 (0.2, 1.2) |  |
| Alcohol history |  |  |  |  | 0.6659 |
| no | 338 | Reference | 0.9 (0.5, 1.4) | 1.0 (0.5, 1.9) |  |
| yes | 53 | Reference | 0.7 (0.2, 2.6) | 0.4 (0.1, 2.2) |  |
| Preoperative Comorbidities | |  |  |  |  |
| Neurological diseases |  |  |  |  | 0.6151 |
| no | 346 | Reference | 0.8 (0.5, 1.4) | 0.9 (0.5, 1.9) |  |
| yes | 45 | Reference | 0.4 (0.0, 4.2) | 0.3 (0.0, 3.2) |  |
| History of cancer |  |  |  |  | 0.9021 |
| no | 350 | Reference | 0.8 (0.5, 1.4) | 0.9 (0.5, 1.7) |  |
| yes | 41 | Reference | 1.0 (0.2, 3.9) | 0.6 (0.1, 5.0) |  |
| Preoperative Laboratory Tests | |  |  |  |  |
| WBC（10^9/L) |  |  |  |  | 0.1964 |
| >10 | 28 | Reference | 0.1 (0.0, 1.3) | 0.7 (0.1, 3.8) |  |
| <=10 | 363 | Reference | 0.9 (0.6, 1.5) | 0.9 (0.5, 1.8) |  |
| Anemia |  |  |  |  | 0.1184 |
| no | 246 | Reference | 1.1 (0.6, 1.9) | 1.8 (0.7, 4.6) |  |
| yes | 145 | Reference | 0.5 (0.2, 1.2) | 0.5 (0.2, 1.2) |  |
| PLT(×10^9/L) |  |  |  |  | 0.7412 |
| Normal PLT | 312 | Reference | 0.7 (0.4, 1.3) | 0.9 (0.4, 1.8) |  |
| Abnormal PLT | 79 | Reference | 1.2 (0.4,3.9) | 1.1 (0.3,3.2) |  |
| ALB(g/L） |  |  |  |  | 0.7094 |
| <35 | 37 | Reference | 1.4 (0.1, 16.0) | 0.5 (0.1, 3.3) |  |
| >=35 | 354 | Reference | 0.8 (0.5, 1.4) | 0.9 (0.5, 1.7) |  |
| SCr(μmol/L) |  |  |  |  | 0.2447 |
| <=100 | 321 | Reference | 0.9 (0.5, 1.5) | 1.1 (0.6, 2.2) |  |
| >100 | 70 | Reference | 0.7 (0.2, 2.4) | 0.3 (0.1, 1.2) |  |
| FIB(g/L) |  |  |  |  | 0.6108 |
| <=4 | 321 | Reference | 0.9 (0.5, 1.5) | 1.0 (0.5, 2.1) |  |
| >4 | 65 | Reference | 0.6 (0.1, 2.5) | 0.5 (0.1, 1.7) |  |
| Anesthesia and Surgical Related Factors | | |  |  |  |
| ASA |  |  |  |  | 0.9778 |
| I -II | 266 | Reference | 0.8 (0.5, 1.4) | 0.8 (0.3, 2.1) |  |
| III | 125 | Reference | 0.9 (0.4, 2.3) | 0.9 (0.3, 2.4) |  |
| Surgical Site |  |  |  |  | 0.0233 |
| Left lung lobe | 159 | Reference | 0.6 (0.3, 1.4) | 2.7 (0.7, 9.7) |  |
| Right lung lobe | 232 | Reference | 1.0 (0.5, 1.8) | 0.5 (0.3, 1.2) |  |
| Thoracoscopic approach | |  |  |  | 0.4174 |
| Thoracoscopy | 332 | Reference | 0.8 (0.5, 1.3) | 0.7 (0.4, 1.4) |  |
| Single-port thoracoscopic | 59 | Reference | 1.3 (0.4, 4.2) | 2.1 (0.5, 9.8) |  |
| Anesthesia Method |  |  |  |  | 0.0629 |
| GEA | 38 | Reference | NA | 1.0 (0.2, 6.6) |  |
| GA | 353 | Reference | 0.8 (0.5, 1.2) | 0.9 (0.5, 1.6) |  |
| Anesthesia Maintenance |  |  |  |  | 0.0710 |
| CIVIA | 47 | Reference | 0.3 (0.1, 1.1) | 0.2 (0.0, 0.9) |  |
| TIVA | 344 | Reference | 1.0 (0.6, 1.6) | 1.1 (0.6, 2.1) |  |
| Surgery duration（min） |  |  |  |  | 0.7963 |
| >=180 | 182 | Reference | 0.8 (0.4, 1.6) | 1.0 (0.4, 2.5) |  |
| <180 | 209 | Reference | 0.9 (0.5, 1.7) | 0.8 (0.3, 1.7) |  |
| Total intraoperative fluid volume (ml) | | | |  | 0.4936 |
| 100 - 1050 | 97 | Reference | 1.7 (0.7, 4.4) | 1.0 (0.3, 3.2) |  |
| 1100 - 1600 | 157 | Reference | 0.7 (0.4, 1.5) | 1.1 (0.4, 2.8) |  |
| 1700 - 4500 | 136 | Reference | 0.6 (0.2, 1.4) | 0.7 (0.2, 2.2) |  |
| Postoperative Pain Management Mode | | |  |  | 0.2553 |
| PCEA | 30 | Reference | 2.1 (0.2, 22.5) | NA |  |
| PCIA | 355 | Reference | 0.8 (0.5, 1.3) | 0.8 (0.5, 1.6) |  |
| Surgeon |  |  |  |  | 0.7530 |
| Surgeon A | 158 | Reference | 0.6 (0.3, 1.4) | 0.7 (0.2, 2.1) |  |
| Surgeon B | 28 | Reference | 0.9 (0.1, 6.8) | 0.2 (0.0, 4.8) |  |
| Surgeon C | 165 | Reference | 1.2 (0.6, 2.5) | 1.5 (0.6, 3.7) |  |
| Surgeon D | 40 | Reference | 0.8 (0.2, 3.2) | 0.6 (0.1, 3.3) |  |

**Abbreviations:**

Abnormal PLT, platelet.>300or<100(10^9/L); SCr, Serum creatinine; FIB, fibrinogen; ASA, American Society of Anesthesiologist Physical Status; GEA, general anesthesia combined with epidural; GA, general anesthesia; TIVA, total intravenous anesthesia; CIVIA, combined intravenous-inhalation anesthesia; PCEA, patient controlled epidural analgesia; PCIA, patient controlled intravenous analgesia; Anemia, hemoglobin <130(g/L) in male or hemoglobin<120(g/L) in female.
